# Ten simple rules for doing a postdoc in pharma

**DOI:** 10.1371/journal.pcbi.1008989

**Published:** 2021-06-03

**Authors:** Jitao David Zhang

**Affiliations:** 1 Pharma Research and Early Development, Roche Innovation Center Basel, F. Hoffmann-La Roche Ltd., Basel, Switzerland; 2 Department of Mathematics and Computer Science, University of Basel, Basel, Switzerland; Dassault Systemes BIOVIA, UNITED STATES

## Abstract

Postdoctoral programs in the pharmaceutical and life science industry offer opportunities for personal and professional development, if you know why to join, what to expect, and how to prepare.

## Introduction

Are you a PhD student or a junior researcher planning for a career in applied research? Are you considering the possibility of joining the pharmaceutical and life science industry as a postdoc? Are you struggling with the lack of information about industrial positions, particularly about their pros and cons compared with academic postdoc positions? You may find this article useful if you have not stopped reading so far.

Among the top 15 pharmaceutical companies ranked by annual sales in 2020, 12 offer dedicated postdoctoral programs (Box 1). Nevertheless, when making career decisions, students and junior researchers are often not aware of these programs. A possible reason is that in contrast to academic postdoc positions, far fewer industrial postdoc positions are available. Consequently, fewer researchers have industrial postdoc experience, which leads to a feedback loop that further reduces the chance for students to learn about such positions from friends, colleagues, and supervisors.

**Box 1**

Many pharmaceutical and life science companies offer postdoc positions. Information about them can be found on the websites of the respective companies as well as on job portals. In case the postdoc position is offered in collaboration with universities or research institutes, job descriptions and information can also be obtained from respective partners.

Below is a list of postdoctoral training programs set up by the top 15 pharma companies ranked by annual sales in 2020. The list is likely incomplete because some companies may have multiple programs, while others may offer similar programs for which I failed to find information.

1. The Roche Postdoc Fellowship (RPF) Programme and Genentech Postdoctoral Programs
2. Novartis Innovation Postdoc Fellowship
3. Merck MRL Postdoctoral Research Fellow Program
4. Glaxo-Smith-Kline Postdoctoral R&D
5. AbbVie U.S. Professional Programs
6. Sanofi’s R&D Postdoctoral Program
7. Pfizer Postdoctoral Program
8. Bristol Myers Squibb Advanced Degree Programs
9. AstraZeneca Postdoc Programme
10. Amgen Postdoctoral Fellows Program
11. Gilead Sciences Research Scholars Programs
12. Novo Nordisk R&D STAR PhD programme, and PhD/postdoc/PharmaD programmes with academic institutes of excellence

In addition to these postdoc programs, many companies provide internship programs for PhD students, for example, Roche Internship for Scientific Exchange (RISE) and Novartis Internship Programs. These and other programs offer PhD students opportunities to learn about industrial research during their study, which may help them make career decisions.

As a cause and a consequence of limited awareness, dedicated discussions about industrial postdoc positions, particularly those in the pharmaceutical and life science industry, are missing in public forums [1]. Meanwhile, great resources are available to help people make career decisions after PhD, among others pieces from the Ten Simple Rules series, including Ten simple rules for choosing between industry and academia [2], Ten simple rules for selecting a postdoctoral position [3], and Ten simple rules for landing on the right job after your PhD or postdoc [4]. This piece attempts to enrich existing literature by raising awareness about industrial postdoctoral programs.

Being aware of these programs is, though, not enough. Based on my admittedly limited interactions with industrial postdocs, including both computational biologists and scientists from other fields, there appear to be two major questions when considering industrial postdoc positions as a career option. The first is whether to apply for them at all, weighing pros and cons compared with academic positions. The second question is, what is special about the application and preparation for an industrial postdoc? This piece suggests ten simple rules to help to-be postdocs address both of these questions.

This article presents a biased view. As a computational biologist working on drug discovery in a pharmaceutical and life science company, I believe that if you have the opportunity to work on an important and motivating topic and if you receive support from good supervisors and other team members, a postdoc position in a company that respects and values research can be rewarding for both your career and your life.

## Pros of doing a postdoc in industry

### Rule 1: Find out whether an industrial postdoc helps you professionally

Ask yourself first whether you want to work for industry. Can you imagine applying your scientific knowledge and personal skills in an industrial setting? Are you comfortable with or excited by the idea of performing applied research to turn discoveries and knowledge into product and profit? While some people believe that science is useless unless it serves the benefit of humankind, others argue that the scientific ideal of objectivity and truth-seeking is eroded by the pursuit of profit. Answering the question of **why** is the first step to decide whether or not to consider an industrial postdoc.

If you are determined to perform industrial research, doing a postdoc there is a good start. Compared with permanent positions, which cannot be permanent in reality, not least because of reorganizations, acquisitions, and mergers, a postdoc position offers an opportunity to experience the working conditions in industry for a predefined, limited period of time.

The competition for postdoc positions is fierce, but the reward is proportionally attractive. During the postdoc, you will enjoy rich resources provided by the company, for instance, the latest technologies, invited talks by top experts, and opportunities to attend scientific conferences and industry workshops. You can connect and collaborate with leading industrial researchers with skill profiles different from those of academics (see also Rule 4 below). If your project is co-supervised by academic researchers or if your team collaborates with groups in universities and research institutes, you can keep and expand your academic connections as well (further discussed in Rule 10). If you seize the chance to make discoveries that improve our understanding of human diseases and reveal how we may treat them, more doors will open for you after the postdoc, including positions that require “industrial working experience,” for which newly graduated PhDs and academic postdocs are (sometimes unreasonably) not eligible.

What if you have not made up your mind about working in industry? A postdoc position offers you both an opportunity to get firsthand experience and more time to reflect before you make decisions.

Even if your long-term goal is an academic career and even if you have reservations about applied research, doing a postdoc in companies may yet pay off. It offers you a comprehensive view of the practical side of your research field and its connections to other disciplines. As an industrial postdoc, besides focusing on your own research, you will be inevitably exposed to other research areas that are at least as exciting and important as your own. Learning about other fields, their importance, their problem-solving approaches, and about the unique contribution of your own research to them, is an important aspect of doing an industrial postdoc. Besides, the postdoc experience may help you identify urgent real-world problems, translate them into valuable scientific questions, and come up with practical and impactful solutions. This advantage is demonstrated by the success of many academic scholars with industrial working experience.

### Rule 2: Find out whether an industrial postdoc helps you personally

People often think that industry offers better benefits and shows more respect for work–life balance compared with academia. Given the precarious positions of postdocs since the start of the Coronavirus Disease 2019 (COVID-19) pandemic [5], such considerations may weigh more in career decision-making. An industrial postdoc position offers a reality check: Do the benefits match the working conditions? Does the work–life balance meet your expectations? The situation differs between research areas, between teams and companies, and between individuals. Doing a postdoc helps you find your own answers.

Apart from financing your life, working as an industrial postdoc also creates, shapes, and expands your personal network, although probably differently than an academic position would do. The industrial network, thanks to the interdisciplinary nature (further elaborated in Rule 3), is likely to consist of more diverse people with regard to their nationality, education, and philosophy of life. It may provide limited help when it comes to publications or grant applications, but does provide you alternative views of work and life, which can be refreshing and thought-provoking. The benefit of such a network is also long-lasting. It can persist when you leave the company after the postdoc. An active and heterogeneous personal network may lead to unexpected job opportunities or new friendships in later stages of your life.

In short, joining industry as a postdoc brings both professional and personal benefits as long as you answered the why question.

## Precautions about doing a postdoc in industry

Rule 3 of Ten simple rules for more objective decision-making [6]: Greater transparency helps in making the right decisions. The advantages of doing an industrial postdoc come with potential challenges and downsides.

### Rule 3: Recognize and understand the new incentive

Industrial and academic research have distinct incentives. While academic research is driven primarily by curiosity and novelty and rewarded mainly by publications, grants, and permanent positions, industrial research puts return of investment at the first place and values practical, applicable innovations that may turn into revenue and profit.

In industrial research, the chance of one discipline producing all the research insights needed to launch a product dwindles to near zero. A complex and valuable product must result from extensive human collaboration—think of the long closing credits following blockbuster films—and cannot be delivered by experts of one discipline. Drug discovery is a prime example for which a long list of disciplines is indispensable: (computational) biology, chemistry, pharmacology, toxicology, computer science, mathematics, statistics, engineering, etc. Successful work in industry is invariably interdisciplinary and collaborative. In contrast, an immortal paper or a distinguished career as a professor can thrive on deep work in one area.

Recognizing and understanding the different incentives in academia and in industry lead us to the importance of teamwork (discussed in Rule 4).

### Rule 4: Communicate and collaborate effectively

Doing an industrial postdoc demands more than excellence in your own field. It requires that you communicate and collaborate with experts of other fields and even influence them. The primary goal is not confined to publishing papers (which is nevertheless often desired, although not necessarily as a first author) or to getting grants (rarely so). You are expected to deliver convincing work that inspires others: scientists, managers, and even investors. For instance, as a computational biologist, you do not stop when you develop new algorithms or models. You strive to make actionable predictions and persuade wet-lab biologists, chemists, and, finally, medical doctors to test them.

Given the incentive for applicable innovations, it may not surprise you that the work of an industrial postdoc is judged not only by novelty, but also by applicability: Is your method robust? Are your results reproducible? Does your work improve the way we discover new drugs? Can we deliver what patients need better and faster based on your research?

Good individual work is necessary but not sufficient to address these questions. You have to make your work understood by colleagues so that they can build on your research to create products in the coming months and years. To achieve this, you have to take a professional attitude and put personal interests behind a common goal, which is not always easy, not only for freshly graduated students. In the past years, academia has become more interdisciplinary, and more PhD students are trained in collaborative environments where teamwork is valued. Nevertheless, the imperative of communication and collaboration often presents a challenge to new postdocs.

### Rule 5: Challenge yourself throughout the postdoc

Working in interdisciplinary teams invites us to reflect about our work and its contribution. You may ask yourself: What is the point of diving so deep in my field if its contribution to the solution of a complex problem is comparably small? Some people see little point and find other jobs when the postdoc project finishes. Others learn why their research matters and are motivated to dive deeper. Yet others spot the needs and gaps on the interfaces between disciplines, develop new techniques and skills, and even create new research areas—they challenge themselves and become giants in the field.

Learning how to constantly learn and apply new things, how to solve complex problems under practical constraints of time and budget, and how to be both a respected scientist and a valuable team player is of prime importance for a postdoc in industry. If you accept the challenge, you may constantly review your work critically and ask yourself: Does my research matter? How can I make it matter even more? How can other people’s work benefit from it?

### Rule 6: Watch out for structural and social pitfalls

Besides the scientific work, structural and social aspects matter, too. Changes may happen to any project or to any team at any time. For instance, a postdoc project was initialized to study a drug candidate X, then the company decided to terminate all projects about X because it failed in clinical trials. In this case, the postdoc project, at least those aspects that require a substantial amount of resources, may have to be implemented differently, however promising the overall scientific project may be. Similarly, in case of a reorganization, a postdoc project may be deprioritized or the postdoc may be expected to work on a different topic from the one with which he/she started.

Structural risks take different forms in small biotechs and in large pharma companies. In a small enterprise, failure of fundraising may mean both the collapse of the company and an abrupt end of a postdoc project. However, the risk of a restructuring is limited. In contrast, large companies may seem financially more stable, but reorganization of teams and reprioritization of projects happen more frequently. Whichever form the structural risk takes, we ignore it at our own peril.

Such scenarios must be unsettling for any researcher, especially if your career depends on it. It is wise to consider these risks before the project starts and to negotiate and implement countermeasures. For example, you and your supervisor can set up the project in such a way that it benefits multiple drug discovery projects, reducing its dependency on the success of a single project. Alternatively, define a plan B to work on a side project that is more resistant against potential structural changes.

Besides a stable environment, postdoc researchers need regular exchange with fellows. Scientific and personal exchange is critical for effective teamwork, for science to thrive, and for mental health. Unfortunately, such exchange does not always come easy for industrial postdocs. The postdoc community is typically small and is often scattered over a large corporate environment. Communication between companies and between company and academia is not only constrained by legal and intellectual property considerations, but also by spatial and organizational separations. Fortunately, communities for postdocs and other junior researchers inside companies are becoming common nowadays so that an industrial postdoc job does not imply working in isolation. More can be done to promote exchange between industrial and academic postdocs.

In brief, if you decide for doing a postdoc in industry, you have to recognize the new incentive, communicate and collaborate effectively while pursuing your own research, challenge yourself constantly, and plan for potential structural risks and changed social structures. Consider this well before you make up your mind.

## Rules to follow once you have made up your mind

### Rule 7: Find the right project and the right supervisor

When you apply for a postdoc opening, make sure that it specifies a research project with well-defined objectives and that a respectable mentor will supervise the project. Not all industrial experts publish many papers: Check patents and conference talks as well.

An ideal postdoc research project in pharma has three properties: It addresses an important question in drug discovery and development; it resonates with you; and it can be executed within the given time and budget. In case of any doubt or ambiguity, try all you can to understand this last detail during the interview.

Your supervisor is ideally a competent scientist who is recognized both inside the company and in the scientific community. He/she should help you design and implement a postdoc project that is both scientifically sound and robust against potential structural changes (Rule 6). Beyond that, the importance of a caring supervisor who respects your ideas, gives you freedom, and supports your development cannot be overestimated. Sometimes a postdoc can have more than one industrial or academic co-supervisor. Make sure that you talk with them during the interview, try to understand what motivates them, and ask yourself whether you connect. Finding the right people to work with is more important than where they work, be it industry or academia.

There is no universal rule for a successful interview. Besides demonstrating your problem-solving skills and how you applied them in your previous work (Rule 3), you may want to prepare examples of how you communicate and collaborate (Rule 4) and how you challenge yourself and encourage others to solve complex problems (Rule 5).

Because postdoc positions are limited and there are usually many outstanding candidates with matching profiles, your application might be rejected. Do not take it personally. Ask for feedback and suggestions if possible.

### Rule 8: Ask questions during and after the interview

During the interview, you can get a mental picture of the project by asking questions to your future supervisor(s) and other team members. They can be about the project: What is the expected outcome in terms of publications, patents, etc.? Are there past experiences and existing solutions in the company that can help with the project? Which concrete applications of the work does the team foresee? The questions can also be about personal and career development: What training opportunities does the company offer? Is there an ombudsman or ombudswoman whom one can turn to in case of conflicts? What are the options after the postdoc? While you may not get the answers to all your questions, asking them helps to reduce the asymmetry of information.

Keep asking questions and upholding communication after you get an offer and enter the job. Each company and each team has its own unwritten “culture code.” Asking explicit questions is an effective way to learn them. It helps to orient yourself and to understand the team. You will soon have a fair idea how team members help each other, how willing they are to help you, and how you can help them in turn. Knowing the background, motivation, and skills of colleagues allows you to find help easier and faster when you need it. This knowledge will also help you strengthen and expand your network by spreading the word to others.

### Rule 9: Balance between staying focused and looking ahead

It is essential to strike a balance between being focused and looking one step ahead. On the one hand, you must focus on your postdoc project and deliver results within defined time and budget constraints. On the other hand, it is wise to prepare a plan for the post-postdoc time, regularly review it, and adjust it accordingly: Am I on the right track with regard to publications, networking, etc.? What skills do I have to learn next? Do not worry if you struggle to find the balance: Given the fast pace in the drug discovery business, we all have to learn all the time.

Although industrial postdocs have bright outlooks in general, it can be dangerous to think of a postdoc contract as a ticket to permanent employment in the same company. My limited observation suggests that while most postdocs find jobs when they finish, only a small proportion get a permanent position within the team they were working in. If you strongly desire to stay in the same team or in the same company, you may improve the odds by collaborating with and helping other postdocs and colleagues, while focusing on your project and delivering impressive results.

### Rule 10: Keep your academic connections

Finally, joining industry does not mean cutting your relationship with academia. It helps to stay in touch with your mentors, peers, and collaborators. If you like the idea, you can share your postdoc experience with your academic supervisor, fellow students, and other scientists, as long as you adhere to the legal and intellectual property rules of the company. By informing others about career options, you expand your personal network in academia. It may even happen that you collaborate with your old lab or other academic partners during and after your postdoc. As the Chinese put it, do not burn the bridge. Build it instead.

Keeping your academic connections also helps if you wish to return to academia at a later time point. Students sometimes worry that their experience in industry may trigger unpleasant questions in future academic job interviews—“Why did you decide to join the industry? Why do you now come back?”. Ultimately, the question is whether an industrial postdoc lowers your chance to get an academic job afterwards. I failed to find any systematic studies to address this question (further research is warranted). Experiences of colleagues and friends suggest that—cautions required!—a capable and resourceful scientist is welcome anywhere, whether he/she has stayed in academia or has switched sides.

Finding the right project and the right supervisor, asking questions, balancing focus and perspective, and keeping academic relations, alas, do not guarantee a perfect start as an industrial postdoc. However, my sincere hope is that these rules help you manage the transition with fewer nasty surprises.

## Conclusions

You are welcome to use the ten simple rules above to inform your decision. Gather more information. Talk with peers, friends, and family members about your ambitions, questions, and worries. Listen to yourself. Make a decision. Go ahead.

At my PhD farewell party ten years ago, knowing that I shall join the industry, a postdoc friend confided me with his prediction: Within 2 or 3 years, I should be either back in academia or looking for a new job. When asked about his reasoning, he touched me with his reply: “For someone like you who is always looking for new problems and challenges, it will soon become boring in industry.”

I am thankful that my friend was both right about me and wrong about industry. There are more than enough problems and challenges in both academia and industry. Research problems in the pharmaceutical and life science industry have direct impact on human life and can be both challenging and fun. An industrial postdoc position invites you and other bright minds to tackle them.
